# In‐MOF‐Derived Hierarchically Hollow Carbon Nanostraws for Advanced Zinc‐Iodine Batteries

**DOI:** 10.1002/advs.202105063

**Published:** 2022-10-01

**Authors:** Lulu Chai, Xian Wang, Yue Hu, Xifei Li, Shaoming Huang, Junqing Pan, Jinjie Qian, Xueliang Sun

**Affiliations:** ^1^ Key Laboratory of Carbon Materials of Zhejiang Province College of Chemistry and Materials Engineering Wenzhou University Wenzhou 325000 China; ^2^ State Key Laboratory of Chemical Resource Engineering Beijing Engineering Center for Hierarchical Catalysts Beijing Advanced Innovation Center for Soft Matter Science and Engineering Beijing University of Chemical Technology Beijing 100029 China; ^3^ Xi'an Key Laboratory of New Energy Materials and Devices Institute of Advanced Electrochemical Energy & School of Materials Science and Engineering Xi'an University of Technology Xi'an Shanxi 710048 China; ^4^ School of Materials and Energy Guangdong University of Technology Guangzhou 510006 China; ^5^ State Key Laboratory of Structural Chemistry Fujian Institute of Research on the Structure of Matter Chinese Academy of Sciences Fuzhou 350002 China; ^6^ Department of Mechanical and Materials Engineering University of Western Ontario London ON N6A 5 B9 Canada

**Keywords:** carbon nanomaterial, hollow morphology, metal‐organic framework, thermal treatment, zinc‐iodine battery

## Abstract

Hollow carbon materials are regarded as crucial support materials in catalysis and electrochemical energy storage on account of their unique porous structure and electrical properties. Herein, an indium‐based organic framework of InOF‐1 can be thermally carbonized under inert argon to form indium particles through the redox reaction between nanosized indium oxide and carbon matrix. In particular, a type of porous hollow carbon nanostraw (HCNS) is in situ obtained by combining the fusion and removal of indium within the decarboxylation process. The as‐synthesized HCNS, which possesses more charge active sites, short and quick electron, and ion transport pathways, has become an excellent carrier for electrochemically active species such as iodine with its unique internal cavity and interconnected porous structure on the tube wall. Furthermore, the assembled zinc‐iodine batteries (ZIBs) provide a high capacity of 234.1 mAh g^−1^ at 1 A g^−1^, which ensures that the adsorption and dissolution of iodine species in the electrolyte reach a rapid equilibrium. The rate and cycle performance of the HCNS‐based ZIBs are greatly improved, thereby exhibiting an excellent capacity retention rate. It shows a better electrochemical exchange capacity than typical unidirectional carbon nanotubes, making HCNS an ideal cathode material for a new generation of high‐performance batteries.

## Graphical abstract

A type of porous hollow carbon nanostraw (HCNS) is in situ obtained by combining the fusion and removal of metallic indium within the decarboxylation process from InOF‐1 nanorod. The HCNS becomes an excellent carrier for electrochemically active species such as iodine with its unique internal cavity and interconnected porous structure on the tube wall to improve the assembled zinc‐iodine batteries.

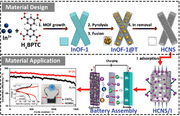

## 1 Introduction

In recent years, the limited fossil energy reserves and the large‐scale use of carbon dioxide (CO_2_) emissions have caused serious ecological and environmental problems. To this end, various countries have successively established strategies to prioritize the development of new energy sources such as solar and wind energy,^[^ ^1^, ^2^, ^3^, ^4^, ^5^ ^]^ and formulated ambitious goals of “carbon peak” and “carbon neutrality” for the future global. Under these circumstances, the electrochemical energy storage (EES) system stands out in the new energy storage with its unique advantages of achieving high efficiency, low cost, high safety, versatility/low dependency on auxiliary infrastructure.^[^ ^6^, ^7^, ^8^ ^]^ At present, halogens (iodine, bromine) and chalcogens (oxygen, sulfur, and selenium) are combined with metal anodes (such as lithium, zinc) to form a variety of attractive EES batteries.^[^ ^9^, ^10^, ^11^ ^]^ Non‐metal elements such as iodine, sulfur, and selenium have the advantages of high safety, fast reaction kinetics, good reversibility, large theoretical capacity, and availability of raw materials. However, poor conductivity and easy ion diffusion in the electrolyte of those non‐metal elements severely limit the improvement of their cycle life. Therefore, it is a promising research direction to develop new types of carbon materials as a universal carrier platform to enhance the conductivity of non‐metal elements and restrain the diffusion of ions for improved electrochemical efficiency.

The currently reported electrode materials such as carbon fiber,^[^ ^12^, ^13^ ^]^ single/multi‐wall carbon nanotubes (CNTs),^[^ ^14^, ^15^ ^]^ and graphite/graphene^[^ ^16^, ^17^ ^]^ have been successively reported as carriers or platforms for small molecular substances. Among them, CNTs with a unique hollow unidirectional tubular structure can be used as a micro‐reaction vessel to confine small molecule active species for electrochemical reactions, which effectively improve the electrochemical performance of electrode active materials. However, the dense tube wall structure and slender tube diameter structure largely restrict the effective diffusion and electron transmission of internal active materials and electrolyte ions.^[^ ^18^ ^]^ Therefore, the development of hierarchically hollow carbon materials (HCMs) has become an urgently needed material for improving new metal secondary batteries.^[^ ^19^, ^20^, ^21^, ^22^ ^]^ For example, Hong et al. reported a green approach to prepare a type of N, B co‐doped hollow porous carbon spheres, exhibiting an improved Na‐/K‐ion storage capacity and cycling life.^[^ ^23^ ^]^ Yang et al. synthesized ultra‐high sulfur‐doped hierarchical porous hollow carbon spheres, which display excellent potassium storage performance due to the reasonable nanostructure and the expansion of the interlayer spacing.^[^ ^24^ ^]^ In 2021, Li et al. developed a self‐templating strategy to prepare the best Ni‐N_5_ active centers combined with ZIF‐8 derived hollow nitrogen‐doped porous carbon (Ni‐N_5_/HNPC). The obtained Ni‐N_5_/HNPC is structurally endowed with abundant hierarchical pores and a large specific surface area, which can provide enough space to accommodate sulfur particles and improve the sulfur utilization rate.^[^ ^25^ ^]^

As an emerging class of porous solids, metal‐organic frameworks (MOFs) are well known as open networks analogous to well‐known zeolites through the coordination and assembly of metal ions or clusters with organic ligands,^[^ ^26^, ^27^, ^28^, ^29^ ^]^ which has aroused widespread interest in the scientific community. MOF materials can form a variety of unique pore structures through decarboxylation and redox between metal oxide particles during pyrolysis, thereby obtaining a variety of porous carbon materials.^[^ ^30^, ^31^ ^]^

**Figure 1:**
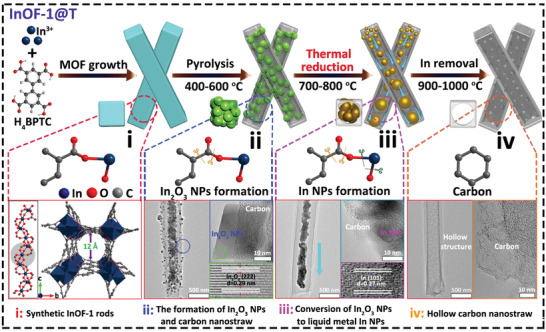
Schematic illustration to obtain InOF‐1 derived hierarchically porous HCNS with their differentiated carbonized stages at different temperatures.

Herein, by virtue to the characteristics of the easy fusion of indium species, the regulation of the ligand decarboxylation process in an indium‐organic framework (InOF‐1) is rationally performed. In this case, the redox reaction of indium oxide nanoparticles (NPs) in the pyrolysis process of InOF‐1 show three obvious structural stages: (i) porous carbon nanorods contain indium oxide NPs; (ii) metallic indium NPs and hollow porous structures are formed inside the carbon rods through redox reactions; (iii) indium NPs aggregate and flow out to form a hollow carbon nanostraw (HCNS) (**Figure** **1**). Electrochemical tests have shown that the MOF‐derived HCNS behaves as a carrier platform for small molecular iodine. And the obtained HCNS can effectively load iodine‐related intermediates as HCNS/I_x_ composite with higher capacity, higher specific power, and longer cycle lifetime than traditional CNTs. Besides, a special cation exchange membrane is utilized to block I^−^ and I_3_^−^ ions, thus effectively avoiding the decrease of battery capacity, and improving its cycle stability. The demonstrated research provides a new frontier material platform for the future development of high‐specific energy secondary batteries such as zinc‐iodine batteries (ZIBs) and lithium‐sulfur batteries.

## 2 Results and Discussion

**Figure 2:**
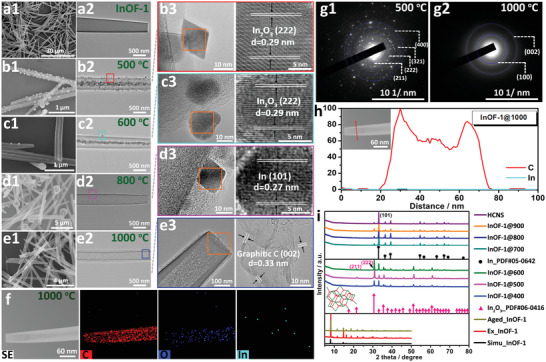
SEM, TEM, and the HR‐TEM images of a) InOF‐1, b) InOF‐1@500, c) InOF‐1@600, d) InOF‐1@800, e) HCNS. f) HAADF‐STEM images and the corresponding EDX elemental mapping for HCNS. SAED patterns of g1) InOF‐1@500 and g2) HCNS. h) Linear scan of element distribution for HCNS. i) PXRD patterns of the precursor InOF‐1 and as‐calcined InOF‐1@T.

Indium nitrate is reacted with biphenyl‐3,3′,5,5′‐tetracarboxylic acid (H_4_BPTC) by a solvothermal method to obtain rod‐like InOF‐1 with a large aspect ratio, whose detailed crystallographic data and structural parameters are listed in Table S1 (Supporting Information). We learn that the In‐based MOF belongs to the tetragonal crystal system, and its nanochannels are formed by In‐O bonds in a 6‐coordinate environment (Figure 1, Figure S1, Supporting Information).^[^ ^32^, ^33^ ^]^ The powder X‐ray diffraction (PXRD) patterns of the as‐synthesized InOF‐1 exhibit five strong diffraction peaks in the 2‐theta range of 8–20° attributed to (111), (200), (211), (220), (202) planes, respectively, indicating a high purity and crystallinity (**Figure** **2**i).^[^ ^34^ ^]^ Raman analysis in Figure S3 (Supporting Information) shows that the observed characteristic peaks in 200–500 cm^−1^ are attributed to metal–oxygen bonds, while the stretching vibrations of C–C (764 cm^−1^, 1164 cm^−1^), O‐H (941 cm^−1^, 1051 cm^−1^), benzene ring (1006 cm^−1^, 1559 cm^−1^, 1608 cm^−1^), O–C═O (1297 cm^−1^, 1438 cm^−1^) from BPTC^4−^ are also clearly observed. Meanwhile, the desolvated samples show well‐maintained structural integrity after thermal activation (Figure S2a, Supporting Information). To investigate the bond information of MOF nanorods, thermogravimetric analysis (TGA) is performed in Figure S2b, Table S2 (Supporting Information), which demonstrates that InOF‐1 with different thermal stability can be divided into four stages: i) the first weight loss of 40–192 °C is attributed to the evaporation of those uncoordinated solvents; ii) the second stage of 192–450 °C is caused by the partial breakage of coordination bonds between In(III) ions and BPTC^4−^ ligands; iii) the third stage of 450–840 °C indicates a complete decomposition of 3‐dimensional framework where InOF‐1 precursors are thermally converted into nanosized In_2_O_3_ particles embedded in the carbonaceous matrix and hollow and tube‐wall porous structures; iv) finally, In_2_O_3_ NPs further undergo redox reactions with carbon particles, and are thermally reduced to metallic indium at 840–1000 °C to form a mesoporous structure. Because indium shows a low melting point of 156.61 °C, the aggregation, fusion, removal, and evaporation of In‐based species occur at this temperature,^[^ ^35^ ^]^ and then the indium flows out after aggregation to leave a hollow morphology.

In order to confirm the above reaction process, we have studied the thermal decomposition behavior of InOF‐1 precursor in 400–1000 °C under Ar by scanning electron microscopy (SEM) and transmission electron microscopy (TEM) images (Figure 2, Figures S4–S12, Supporting Information). Before carbonization, the rapid solvothermal reaction of In(III) and H_4_BPTC leads to the formation of rod‐like morphology with a diameter of ≈700 nm (Figure 2a, Figure S6, Supporting Information). After that, SEM and TEM images of InOF‐1@400 corroborate that a few nanosized particles are evenly deposited on the surface at 400 °C. In this case, InOF‐1 structure is gradually carbonized and decomposes to form In_2_O_3_ NPs. Meanwhile, the elements of C, O, In are well distributed on a single rod in the EDX mapping (Figure S9, Supporting Information). To observe the formation and composition of HCNS, high‐resolution TEM (HR‐TEM) images are obtained from all samples (Figure 2, Figures S10 and S11, Supporting Information). At 500 °C, In_2_O_3_ NPs begin to aggregate on the external wall, while abundant particles gather in the middle of InOF‐1@500 in Figure 2b, in which HR‐TEM images confirm the obvious spacing of 0.29 nm assigned with the (222) plane of indium oxide in agreement with the selected area electron diffraction (SAED) image (Figure 2g1). Moreover, the formed In_2_O_3_ originates mainly from the rupture of In–O chains, and the graphitic carbon derives from the in situ carbonization of organic ligands. During 600–700 °C, In_2_O_3_ NPs are further reduced into large particles, while the aggregation of metallic indium promotes the generation of hollow morphology and porous structure of the tube wall (Figure 2c, Figure S10, Supporting Information). Due to the influence of surface tension, In NPs aggregate and flow out of the nanorod through the fusion process, which easily volatilizes to release indium‐rich vapor to obtain neat HCNS in 800–1000 °C. It is highly consistent with SEM, TEM, and HR‐TEM images for InOF‐1@800/900/1000 (Figure 2d,e, Figure S11, Supporting Information). Meanwhile, a remaining particle in InOF‐1@800 reveals the spacing of 0.27 nm indexed to the (101) plane of indium. At 1000 °C, the hollow HCNS exhibits an inter‐lattice distance of an amorphous carbonaceous framework of 0.33 nm corresponding to the (002) plane of graphitic carbon confirmed by the SAED image with the characteristic ring diffractions (Figure 2g2). Furthermore, the elemental mapping and line scan spectra of HCNS by the high‐angle annular dark filed scanning‐TEM (HAADF‐STEM), demonstrating that C atoms are homogeneously distributed, while only the residual In atoms are observed (Figure 2f,h). The PXRD patterns further confirm the composition and structure of InOF‐1@T in Figure 2i, where all diffraction peaks of InOF‐1@400/500/600 belong to cubic In_2_O_3_ (PDF#06‐0416), revealing that In‐based MOFs can be easily converted to In_2_O_3_ NPs during the low‐temperature pyrolysis. However, as it rises up to 700–1000 °C, the (222) plane intensity of In_2_O_3_ is gradually weakened and fully converted into indium in InOF‐1@700/800/900/1000 with the emerging (101) plane (PDF#05‐0642).

**Figure 3:**
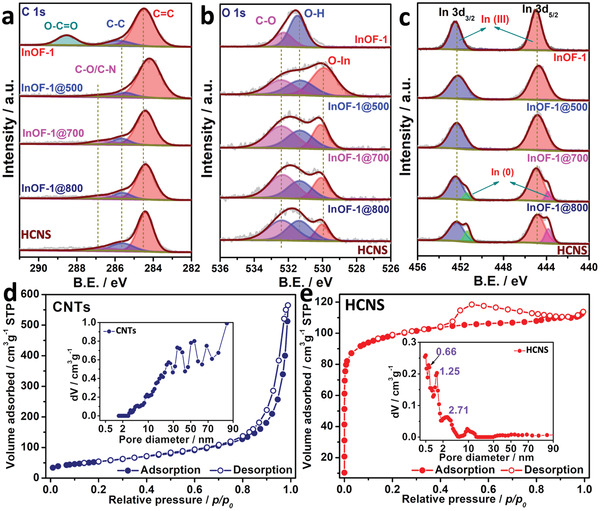
The deconvoluted XPS spectra of a) C 1s, b) O 1s, c) In 3d of InOF‐1@T. N_2_ isotherms and PSD curves of d) CNTs and e) HCNS.

We have further probed the electronic structures in the InOF‐1 and their annealed samples using X‐ray photoelectron spectroscopy (XPS) and Raman spectra, respectively. The C, O, and In as well as the residual N atoms are evidenced by the XPS survey in **Figure** **3**, Figures S13 and S14 (Supporting Information). First, the deconvoluted C 1s spectrum of InOF‐1 reveals three well‐defined peaks at 284.5, 285.9, and 288.5 eV corresponding to the C═C, C–C, and –COO, respectively, which stem from the aromatic carbons of BPTC^4−^ ligands in Figure 3a. In addition, there is a small amount of N atoms in InOF‐1 fitted into the C–N of 400.1 eV and the –NO_3_ of 406.0 eV from the solvent and nitric acid. At different temperatures, the chemical composition in the C 1s spectra differs where the –COO bonds gradually disappear, while new C‐O/CN bonds emerge at 286.4 eV to indicate the occurrence of decarboxylation.^[^ ^36^ ^]^ The chemical states of the residual O atoms are featured with two types of oxygen species: the C–O groups (533.2 eV) and the O–H groups (531.8 eV) in the carbon skeleton (Figure 3b). The O 1s peaks at 530.1 eV are attributed to lattice oxygen of the In_2_O_3_ phase. During heating, as the percentage of In‐O bond gradually decreases, it indicates that In_2_O_3_ can be thermally reduced, and then forms the accumulation and removes volatilization of indium consistent with the PXRD results. The high‐resolution XPS spectra of In 3d show two prominent peaks corresponding to In 3d_3/2_ and In 3d_5/2_ (Figure 3c), in which the peak at 444.6 eV corresponds to In 3d_5/2_ of In(III) ion, while the one at 452.3 eV is to In 3d_3/2_.^[^ ^37^, ^38^ ^]^ However, two distinct In(0) peaks at 443.7 and 451.4 eV for InOF‐1@800 are observed, confirming that In_2_O_3_ species can be thermally reduced in the MOF‐derived carbon materials. Furthermore, Raman spectra display two peaks at 1345 and 1585 cm^−1^ referred to D‐band of structural disorder and the G‐band of sp^2^‐hybridized carbon in Figure S15 (Supporting Information), respectively. It is learned that the calculated *I*_D_/*I*_G_ ratios of InOF‐1@T (*T* = 500–1000) samples range from 0.81 to 1.01, implying the enhanced defects induced by surface oxidation.

The porous structure is first determined by N_2_ isotherms, in which the desolvated InOF‐1 shows a type‐I sorption isotherm with micropores of 0.5–1.8 nm with the BET and Langmuir surface area of 870.9 and 937.8 m^2^ g^−1^, respectively (Figure S16, Supporting Information). On the other hand, the hysteresis loop in the relative pressure of 0.40–1.00 signs the existence of mesoporous structures for InOF‐1@T (*T* = 400–1000) while retaining the partial micropores of the original MOFs (Figures S17 and S18, Supporting Information). The specific surface areas, total pore volumes, and micropore volumes are summarized in Table S3 (Supporting Information). In Figure 3d, commercial CNTs exhibit the type‐IV sorption isotherms with a relatively smaller BET/Langmuir surface area (192.9/195.8m^2^ g^−1^). And the pore size distribution (PSD) of CNTs is mainly distributed between 3 and 100 nm, which provides a platform for effective encapsulation of small molecules. However, N_2_ isotherms in Figure 3e further prove the surface area and porous structure of HCNS, which owns a characteristic type‐IV isotherm, indicating the presence of the micropores of the MOF precursor and rich mesopore/macropore after pyrolysis. For its PSD curve (Figure 3e inset), there are micropores of 0.66 and 1.25 nm, and mesopores of 2.71 and 9.98 nm well comparable to the diameter of I^−^ (0.21 nm), I_2_ (0.27 nm), and I_3_^−^ (0.58 nm).^[^ ^39^, ^40^ ^]^ The above method consolidates that the as‐pyrolyzed HCNS as a porous template can substitute CNTs as a carrier platform for electrode materials and active catalysts, which encourages us to further investigate the effective encapsulation of iodine‐based molecules.

**Figure 4:**
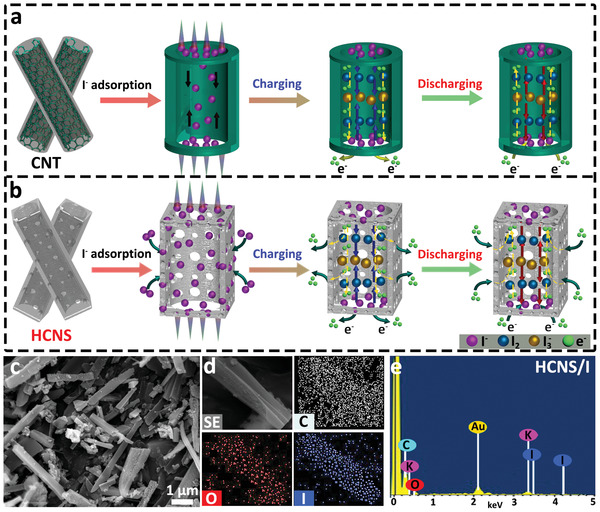
Schematic illustration of charge storage mechanism in a) CNTs and b) HCNS during charging/discharging process. c) SEM image, d) elemental mappings, and e) EDX spectrum of HCNS/I_0.5_.

The advantages of the hollow and porous structure of HCNS in comparison with the commercial CNTs are schematically depicted in **Figure** **4**a,b. It is well known that the CNT with a unique hollow tubular structure loads small molecular substances by providing enough internal space, which can buffer the material in volume effect during the charging/discharging process. However, the dense tube wall limits the ability of small molecules to be captured by the carbon layer, resulting in small iodide ions that can only be carried in the tube and undergo electrochemical exchange through a longitudinal shift. Therefore, this confinement prolongs the transmission path of electrons/ions, which reduces the rate performance and the utilization efficacy of active materials, thereby sacrificing the energy and power density of the ZIBs to a certain extent. In contrast, HCNS not only owns a similar hollow structure but also retains the microporous structure of MOF precursor and the formed meso/macropores. The hierarchical pores can synergistically store more charge active sites, which also greatly shorten the rapid transport path of mass and electron. SEM image, element mapping, and the corresponding EDX spectrum (Figure 4c–e, Table S4, Supporting Information) confirm that iodide ions have been successfully encapsulated into HCNS with uniform adsorption into the entire hollow carbon. However, the corresponding C, N, O, and I contents of CNT/I_0.5_ are successfully obtained by the elemental mappings and EDX analysis, where only 2.61 wt% of iodide ions are encapsulated in the CNT due to the dense carbon wall (Figures S19 and S20, Supporting Information).

**Figure 5:**
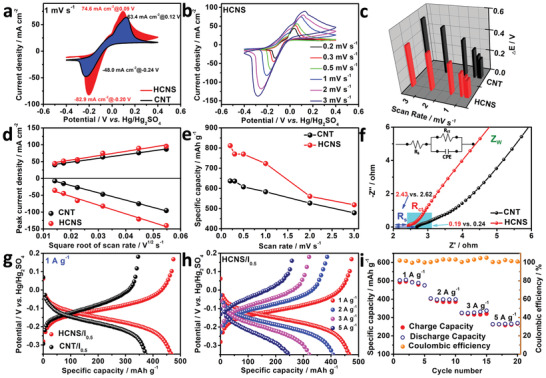
a) CV curves at 1 mV s^−1^ of CNT and HCNS. b) CV curves of HCNS. c) Peak potential separation values. d) The function of *I*_p_ and *v* ^1/2^. e) The specific capacity from the CV curves. f) Nyquist plots. g) Voltage profiles of HCNS/I_0.5_ at 1 A g^−1^. h) Voltage profiles of CNT/I_0.5_ and HCNS/I_0.5_. i) Rate capability of HCNS/I_0.5_ at various current densities.

On the other hand, cyclic voltammetry (CV) curves in **Figure** **5**a, Figure S21 (Supporting Information) show two obvious peaks corresponding to the I^−^/I_3_^−^ redox pair. By comparing the positions of the oxidation peak (*E*_O_) and reduction peak (*E*_R_), the degree of peak potential separation (Δ*E* = *E*_O_–*E*_R_) of HCNS is decreased by 63.6 mV than CNT catalyst at 1 mV s^−1^, indicating that HCNS improves the electrochemical activity and reversibility (Figure 5c). Meanwhile, the fitting results of the redox peak current (*I*_p_) and the square root of the scan rate (*v* ^1/2^) exhibit a good linear relationship that is mainly caused by the diffusion‐controlled intercalation/deintercalation (Figure 5d). Besides, compared to CNT (583 mAh g^−1^), HCNS reaches an outstanding capacity at 1 mV s^−1^ (723 mAh g^−1^), and its capacity drops sharply as the scan rate increases (Figure 5e). The Nyquist plots in Figure 5f exhibit that the charge‐transfer resistance of HCNS (*R*_ct_, 0.19 Ω) is obviously smaller than the CNT (0.24 Ω) as evidenced by a smaller semicircle, due to the high graphitization degree, the rich porous structure, and unique hierarchical hollow structure. Meanwhile, HCNS reveals a smaller series resistance (*R*_s_ = 2.43 Ω) and a higher slope of Warburg impedance (*Z*_W_) than those of CNT, which means that the catalyst has better conductivity to accelerate the electron transmission rate. Furthermore, the constant current charge and discharge (GCD) characteristics of different iodine content loaded into the nanostraws as the cathode are depicted in Figure S22 (Supporting Information). With the increase in the iodide ion content of 0.10–0.75 m, the discharge specific capacity increases from 119.1 to 799.5 mAh g^−1^. However, when it is 0.75 m, the balance of adsorption and dissolution of excessive iodine species in the electrolyte is disrupted as a result of the soluble iodine formed. In Figure 5g, the specific discharge capacity of the CNT/I_0.5_ catalyst (374.0 mAh g^−1^) is slightly lower than that of HCNS/I_0.5_ (466.2 mAh g^−1^) at 1 A g^−1^. The GCD curves of Figure 5h show that when the current densities are 1, 2, 3, and 5 A g^−1^, the specific discharge capacities of HCNS/I_0.5_ are calculated to be 466.2, 406.6, 320.9, and 247.2 mAh g^−1^, respectively. The specific discharge capacity of CNT/I_0.5_ decreases significantly at different current densities (Figure S23, Supporting Information). According to the rate performance test in Figure 5i, the capacity retention rates of HCNS/I_0.5_ after 5 cycles are 100% (475.4 mAh g^−1^) and 101% (269.4 mAh g^−1^), respectively. This excellent rate performance is closely related to the unique hollow structure and rich porous structure of HCNS, which can well encapsulate the iodine species so that the redox reaction of iodine occurs in the channels and the micro/mesopores of the confined space, which ensures that the adsorption/dissolution of the iodine species facilitates an equilibrium.

**Figure 6:**
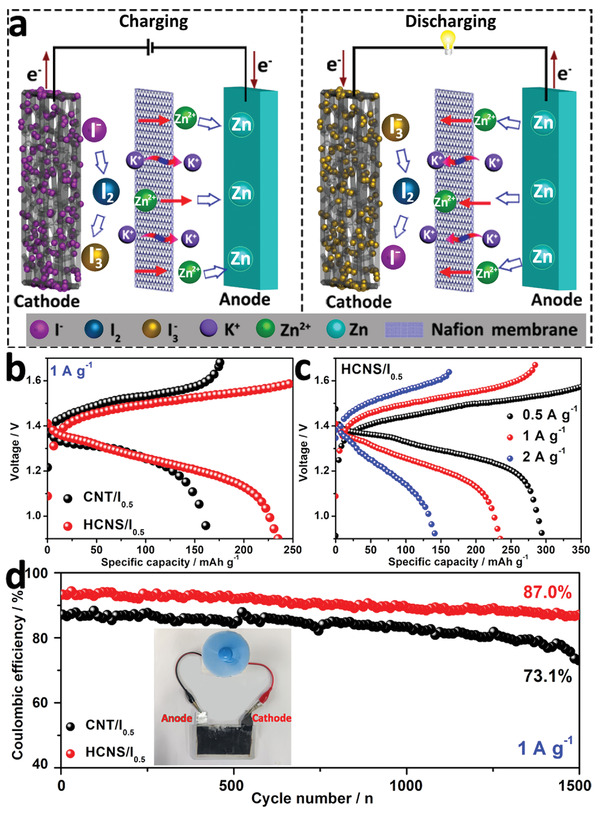
a) Schematic operation mechanisms of a ZIB with a HCNS/I_0.5_ as cathode and Zn flake as anode material. b) Voltage profiles of HCNS/I_0.5_ and CNT/I_0.5_ at 1 A g^−1^. c) Voltage profiles of HCNS/I_0.5_ under different current densities. d) Cycling stability of at 1 A g^−1^, the inset in (d) shows an electric fan motor driven by the assembled device.

To evaluate the practical ZIB performance composed of HCNS/I_0.5_ and CNT/I_0.5_ as the cathode and zinc sheet as the anode, we have conducted GCD tests on the full battery (**Figure** **6**b,c, Figure S24, Supporting Information), respectively. The commercial CNT catalyst exhibits a lower discharge capacity (177.4 mAh g^−1^) and a large polarization voltage at 1 A g^−1^ (Figure 6b). Surprisingly, the assembled ZIB with HCNS/I_0.5_ provides the maximum discharge capacity of 295.7 mAh g^−1^ at 0.5 A g^−1^, while it shows 234.1 and 143.3 mAh g^−1^ from 1 to 2 A g^−1^, respectively (Figure 6c). Meanwhile, the modified cation membrane can effectively and selectively shield I^−^ and I_3_^−^ ions to prevent the crossover of iodine species and maintain stability in the electrolyte. Compared with CNT/I_0.5_, the long‐term cycling stability shows a high Coulombic efficiency (1st, 94%; 1500th, 87%) for the HCNS/I_0.5_ based ZIB at 1 A g^−1^ (Figure 6d). In addition, we have further assembled a soft‐packed ZIB to successfully empower an electric fan in Figure 6d inset (Movie S1, Supporting Information). In this context, the assembly of a two‐electrode ZIB can maintain high capacity and energy efficiency, as well as excellent cycle stability. In this case, HCNS exhibits higher electrochemical performance, higher capacity, and excellent stability than CNTs (Figure 6a), which can be ascribed to four factors: (i) The rich porous structure and can effectively capture and adsorb iodine substances; (ii) The unique hierarchical hollow structure can provide a convenient transmission channel for electron and reactants, which can buffer the volume effect of redox substances; (iii) The formed carbon skeleton improve the electronic conductivity to effectively improve the charge transfer and the high utilization rate of electrode materials; (iv) The unique Nafion cation exchange membrane can not only effectively block I^−^ and I_3_^−^ anions, but also maintain a high concentration of the two anions in the cathode area, thereby effectively avoiding the decrease in specific capacity and enhancing the cycle stability of electrode materials.

## 3 Conclusion

In summary, we have successfully prepared a low‐melting, oxygen‐philic indium‐based MOF nanorod by combining the particularity of metallic indium. With the change of carbonization temperature, the InOF‐1 nanorods will gradually form metal In NPs, which will be thermally removed from the internal tube through the fusion process to form a carbon nanostraw with a hollow structure. As a new type of micro‐reaction vessel, the hierarchically porous HCNS catalyst exhibits higher specific capacity and excellent cycle stability than typical CNTs, mainly due to the high specific surface area, hollow structure, and controlled pore structure. Through this report, we have demonstrated that MOFs are regarded as a type of promising precursors and/or templates for the facile synthesis of hollow and porous carbon nanomaterials with advanced functions, which will further provide unlimited possibilities for the precise construction of a catalyst with a precise structure.

## 4 Experimental Section

### Preparation of InOF‐1 ([In_2_(OH)_2_(BPTC)]⋅6H_2_O)

The synthesis of InOF‐1 was first slightly modified by the following reported methods.^[^ ^29^ ^]^ The In(NO_3_)_3_·xH_2_O (0.10 mmol, 30 mg), H_4_BPTC (0.05 mmol, 15 mg), and CTAB (0.01 mmol, 5 mg) were weighted and 35 mL pressure‐resistant tube (Beijing Synthware Glass, Inc.) was added with them. The solvents of DMF (3 mL), H_2_O (3 mL), HNO_3_ (0.1 mL), and TEA (0.1 mL) were injected into the pressure‐resistant tube using a matching pipette. The mixture was sonicated for 10 min to fully dissolve the metal salt and the ligand, held at 140 °C for 30 min, and then cooled to 25–40 °C. InOF‐1 nanorods were collected by centrifugation multiple times at a centrifugation speed of 10 000 rpm by using DMF and EtOH distribution, and then dried under vacuum at 85 °C overnight. By calculation, 20 mg MOF powder was obtained with about 57.4% yield based on the initial organic ligand. The yield of the InOF‐1 was calculated according to the following equations:

(1) $$M_{\mathrm{InOF}-1}=696.41g\mathrm{mo}l^{-1}$$

Calculate the amount of InOF‐1 that should be theoretically obtained based on the amount of H_4_BPTC:

(2) $$m_{T}=n\times M_{\left(\mathrm{InOF}-1\right)}=0.05\mathrm{mmol}\times 696.41g\mathrm{mo}l^{\left(-1\right)}=34.82\mathrm{mg}$$

(3) $$\gamma =\frac{m_{A}}{m_{T}}\times 100\%=\frac{20\mathrm{mg}}{34.82\mathrm{mg}}\;\times 100\%=57.4\%$$

### Preparation of InOF‐1 Annealed at Various Temperatures (InOF‐1@T)

The above‐synthesized InOF‐1 (200 mg) was used as the precursor and transferred into a CVD tube furnace, calcined to various temperatures (from 400 to 1000 °C) for 2 h with a ramp of 20 °C min^−1^ under Ar atmosphere flow. After naturally cooling down to room temperature, the products were collected and marked as InOF‐1@T where *T* is the carbonization temperature, according to the same calcination conditions, respectively. Among them, HCNS (≈70 mg) was obtained in ≈35% yield based on InOF‐1. In addition, these rod‐like InOF‐1 and HNCS materials with hollow porous structure can be reproduced well in this experiment.

### The Pretreatment for HCNS

The obtained HNCS material was added to a mixed solution containing 5 mL of HNO_3_, 5 mL of H_2_SO_4_, and 20 mL of deionized water, and was reacted at 800 rpm and 50 °C for 12 h. Finally, the products were centrifuged with distilled water until the pH was neutral and dry in a vacuum oven at 85 °C. The purpose of the above operation was to remove the elemental indium and provide conditions for storing small molecules.

### Preparation of HCNS/I_x_ Composites

To prepare the KI‐supported carbon materials, different amounts of KI (0.1 , 0.2, 0.4, 0.5, and 0.75 m) were added to deionized water, and the mixtures were sonicated at room temperature until the KI was fully dissolved. Next, 20 mg of acid‐treated *HCNS*@1000 was added to the above solution by sonication for 2 h to adsorb KI. Finally, it was centrifuged with deionized water and dried in an oven at 60 °C for 12 h to remove residual water.

## Conflict of Interest

The authors declare no conflict of interest.

## Supporting information

Supporting Information

Supplemental Movie 1

## Data Availability

The data that support the findings of this study are available in the supplementary material of this article.
