# Supplementary material for: In‐MOF‐Derived Hierarchically Hollow Carbon Nanostraws for Advanced Zinc‐Iodine Batteries
Source: Adv Sci (Weinh). 2022 Oct 1;9(33):2105063. doi: 10.1002/advs.202105063 (PMC9685461; doi:10.1002/advs.202105063)
Supplement: Supplementary file 1 — Supporting Information [file ADVS-9-2105063-s001.pdf]

ADVANCED SCIENCE
Open Access

Supporting Information

for *Adv. Sci.*, DOI 10.1002/advs.202105063

In-MOF-Derived Hierarchically Hollow Carbon Nanostraws for Advanced Zinc-Iodine Batteries

Lulu Chai, Xian Wang, Yue Hu, Xifei Li, Shaoming Huang, Junqing Pan, Jinjie Qian* and Xueliang Sun**

WILEY-VCH

ADVANCED SCIENCE Open Access

Supporting Information

for *Adv. Sci.,* DOI: 10.1002/advs.202105063

In-MOF-derived hierarchically hollow carbon nanostraws for advanced zinc-iodine batteries

Lulu Chai, Xian Wang, Yue Hu, Xifei Li, Shaoming Huang, Junqing Pan, Jinjie Qian* and Xueliang Sun**

Supporting Information

In-MOF-derived hierarchically hollow carbon nanostraws for advanced zinc-iodine batteries

Lulu Chai, Xian Wang, Yue Hu, Xifei Li, Shaoming Huang, Junqing Pan, Jinjie Qian* and Xueliang Sun**

Experimental Details

Chemicals and materials.

Unless otherwise specified, chemicals are reagent grade and used without processing. Indium nitrate hydrate ($In(NO_3)_3 \cdot xH_2O$, 99.9%, Aladdin), Cobalt(II) acetate tetrahydrate ($Co(CH_3COO)_2 \cdot 4H_2O$, 99.5%, Aladdin), biphenyl-3,3',5,5'-tetracarboxylic acid (H_4BPTC, 98.0%, Jinan Henghua Technology Company), hexadecyl trimethyl ammonium bromide (CTAB, 99%, Aladdin), polyvinylpyrrolidone (PVP, ~58000, Aladdin), N,N-Dimethylformamide (DMF, 99.5%, Aladdin), N-Methylformamide (NMF, 99%, Aladdin), de-ionized water (18 MΩ), triethylamine (TEA, 99%, Aladdin) Nitric acid concentrated solution (HNO_3, 70%, Aladdin), and ethanol (EtOH, 95%, Aladdin).

Materials Characterization

The microscopic and nanostructured morphologies of all samples are characterized by scanning electron microscopy (SEM, JEOL JSM-6700F, 10 kV), transmission electron microscopy (TEM, FEI Tecnai F20, 200 kV), high-resolution transmission electron microscope and energy dispersive X-ray spectroscopy analyses (HR-TEM and EDS, JEOL JEM-2100F, 200 kV). The powder X-ray diffraction (PXRD) patterns are collected on a Bruker D8 Advance at 40 kV and 40 mA with Cu Kα radiation (λ=0.154 nm). Thermogravimetric analysis (TGA) is implemented under a flowing N_2 atmosphere by using a NETZSCH STA 449C unit. Raman spectrometer is investigated on LabRAM HR Evolution from the 532 nm line of an Ar-ion laser. X-ray photoelectron spectroscopy (XPS) is recorded on a Thermo Scientific ESCALAB 250. Fourier transform infrared spectroscopy (FT-IR) spectra are carried on in the model of PerkinElmer Frontier MIR. N_2 adsorption/desorption isotherms are used to characterize the determine specific surface areas and pore distribution of samples based on the Brunauer-Emmett-Teller method (BET, Micrometrics ASAP 2020 system).

Electrochemical measurements

A three-electrode system consisting of a catalyst supported on a 1×1cm^{-2} carbon cloth (CC) as the working electrode, Hg/Hg_2SO_4 electrode as the reference electrode and platinum sheet as the counter electrode, was performed to test its electrochemical performance by CHI760E workstation (CH Instruments, Shanghai) and LANHE battery-testing instrument (Wuhan Instruments) respectively. 0.5 M H_2SO_4 was employed as the electrolyte of a three-electrode system. To fabricate the iodine cathode, the **HCNS/I$_x$** composite material (5 mg) was pulverized and dispersed in ethanol (500 μL) and Nafion (5%, 50 μL) solution, which was ultrasonicated for 2 hours to form a uniform slurry. Subsequently, the catalyst was evenly dropped onto the surface of the carbon cloth and allowed to dry naturally. All the Galvanostatic charge-discharge (GCD) curves were tested on the LANHE multichannel battery test instrument. The operating voltage window is -0.5-0.2 V. Moreover, cyclic voltammetry (CV) curves from -0.5 to 1.5 V (*vs* Hg/Hg_2SO_4) at different scan rates were carried out using a CHI760E workstation. All operations were observed at room temperature.

Assembly test of zinc-iodine battery

For the iodine cathode, the **HCNS/I$_{0.5}$** on CC with an area of 1×1 cm^{-2} was identified as cathode. For the anode, a zinc flake with a thickness of 20 μm was selected. The mixed solution of 0.5 M $ZnSO_4$ plus 0.5 M H_2SO_4 was designated as the aqueous electrolyte of the battery. The GCD curves were obtained by LANHE system at different current densities at room temperature. During GCD tests, the battery was operated at different current densities with designed charge and discharge cut-off voltage. Prior to full-battery fabrication, the cathode was electrochemically activated in a half cell, this step is critical to circumvent the large irreversible capacity and the low Coulombic efficiency (CE).

Crystal Data and Refinement Results

Table S1. Single crystal X-ray data for rod-like InOF-1.

**Compound**	**InOF-1**
Chemical formula	$C_{16}H_{20}In_2O_{16}$
Formula mass	696.41
Crystal system	tetragonal
Space group	$I4_122$
a (Å)	15.5665(6)
b (Å)	15.5665(6)
c (Å)	12.3201(1)
α (°)	90
β(°)	90
γ(°)	90
Unit cell volume (Å^3)	2985.36
Temperature (K)	293(2)
Z	8
F(000)	1328
No. of reflections measured	11697
R_{int}	0.024
Final R_1 values (I>2σ(I))	0.016
Final wR (F_2) values (I>2σ(I))	0.048
Flack parameter	0.26(4)

Additional Structure Images

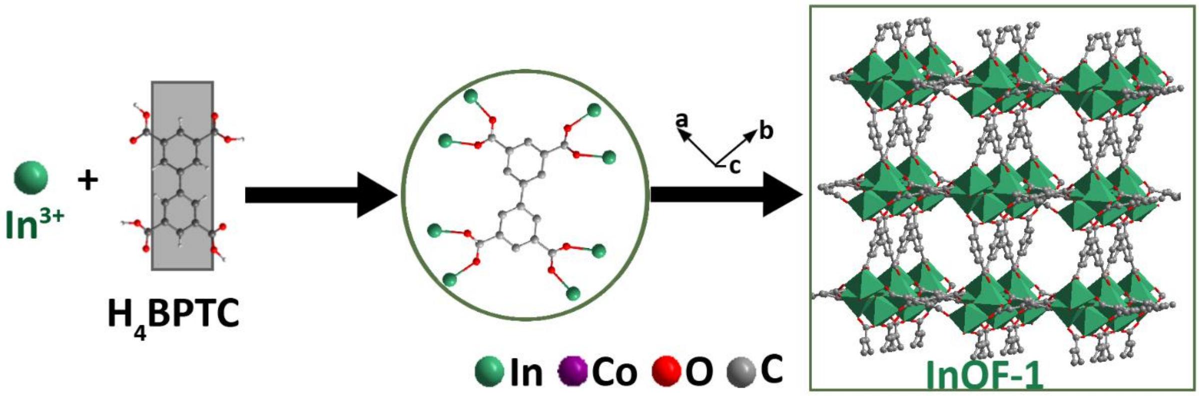

Figure S1. The synthesis and crystal structure of **InOF-1**.

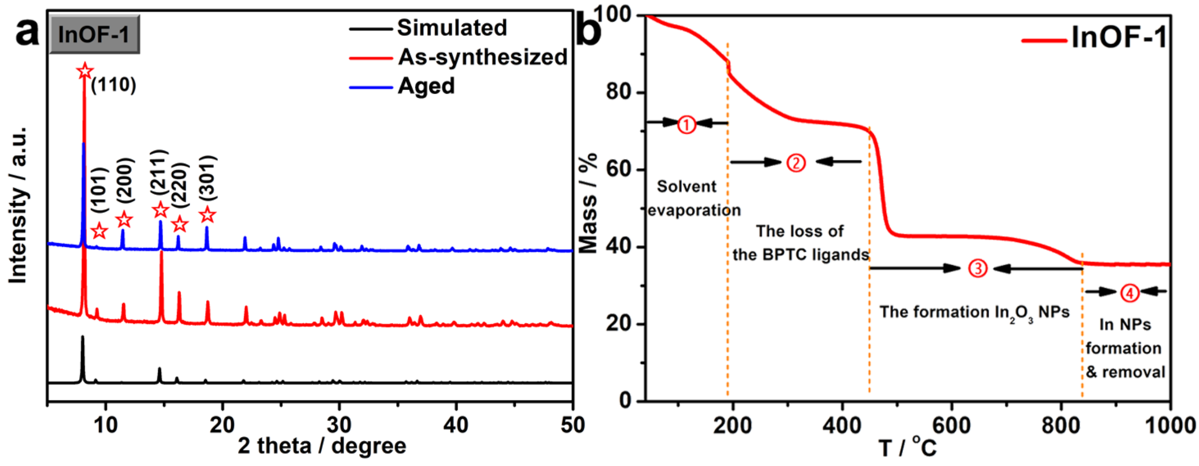

Figure S2. (a) PXRD patterns of the as-synthesized, activated and simulated **InOF-1**. (b) TGA curves of **InOF-1**.

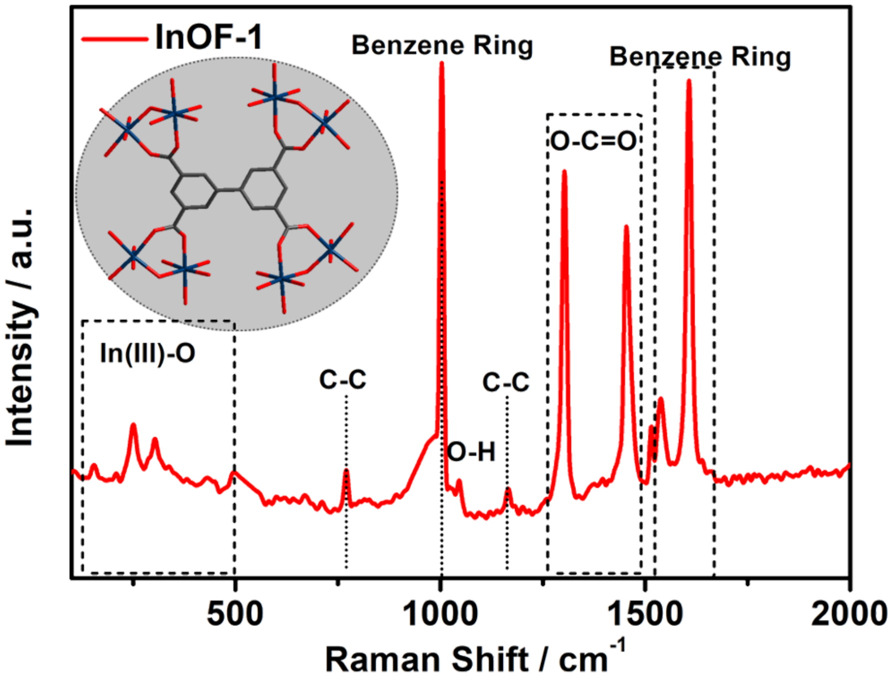

Figure S3. Raman spectra of **InOF-1** crystals.

Table S2. The bond length and bond energy of the chemical bonds in the structure

Chemical bond	Bond length (Å)	Bond energy (kJ mol^{-1})
C-O	1.275	326
C-C	1.490	332
In-O	2.152	320.1±41.8
C=C	1.388	615
C=O	1.255	728

Additional Schematic Diagrams

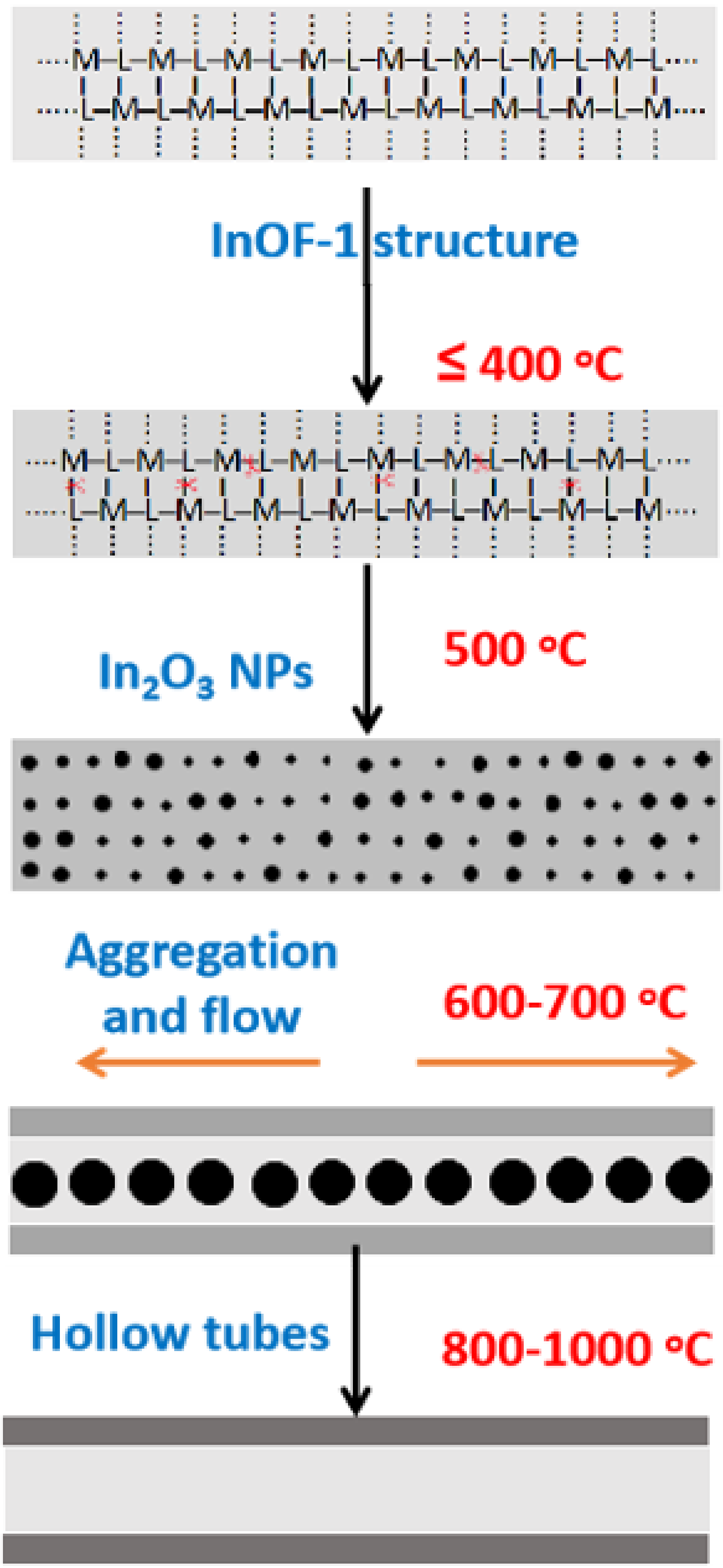

Figure S4. Schematic diagram of carbonization phenomenon of **InOF-1** at different temperatures.

Additional SEM, TEM, EDX Images

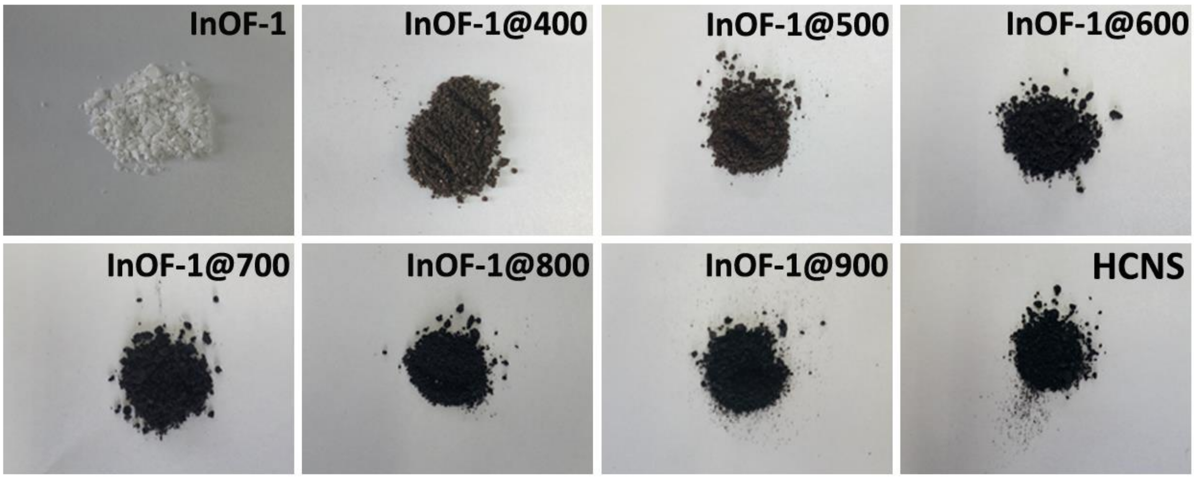

Figure S5. Digital photographs of **InOF-1** and its derivatives of **InOF-1@T** calcined at different temperatures.

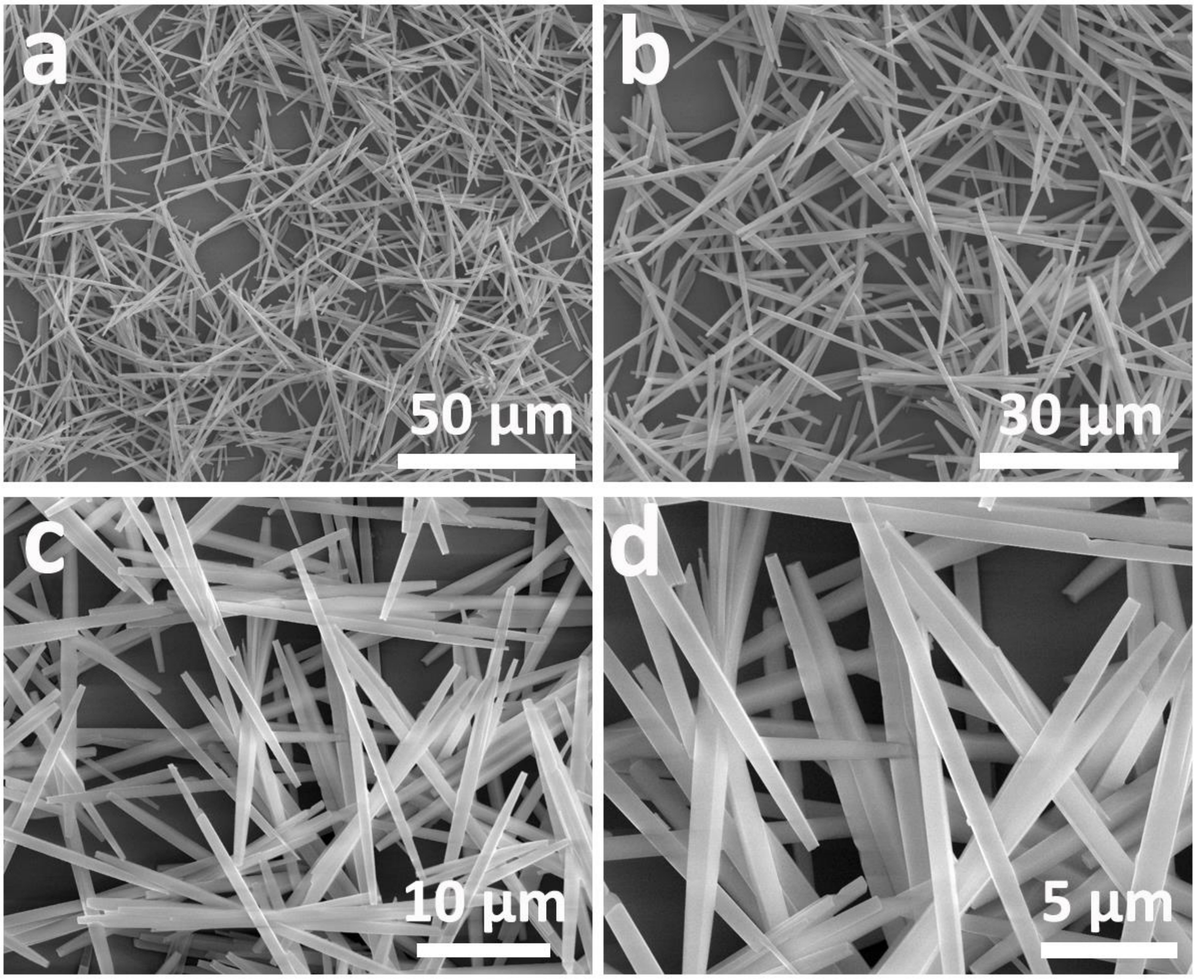

Figure S6. SEM images at different resolutions of **InOF-1**.

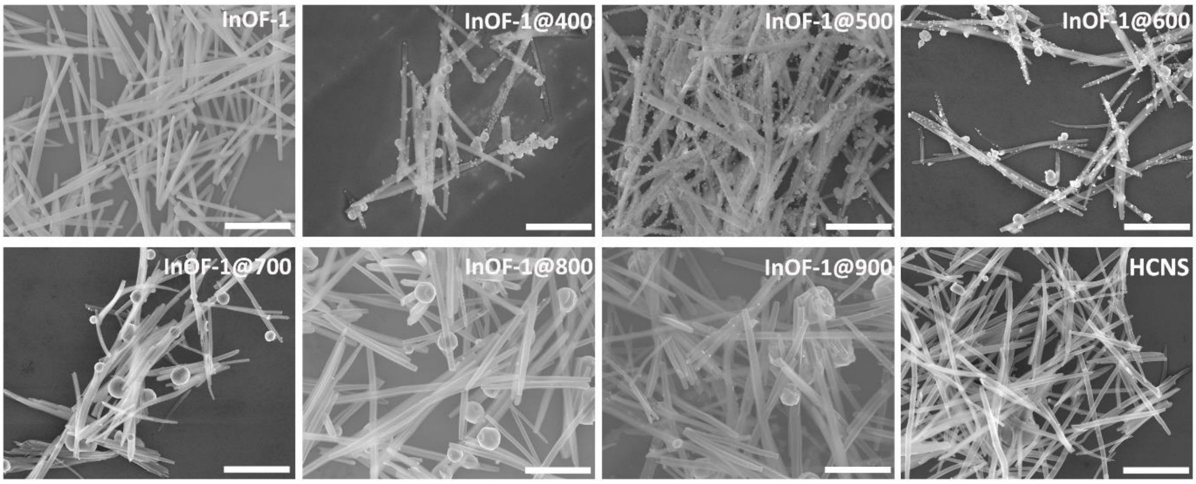

Figure S7. SEM images of initial **InOF-1** nanorods and its derivatives annealed at various temperatures of **InOF-1@T** (T=400-1000), scale bar: 5 μm.

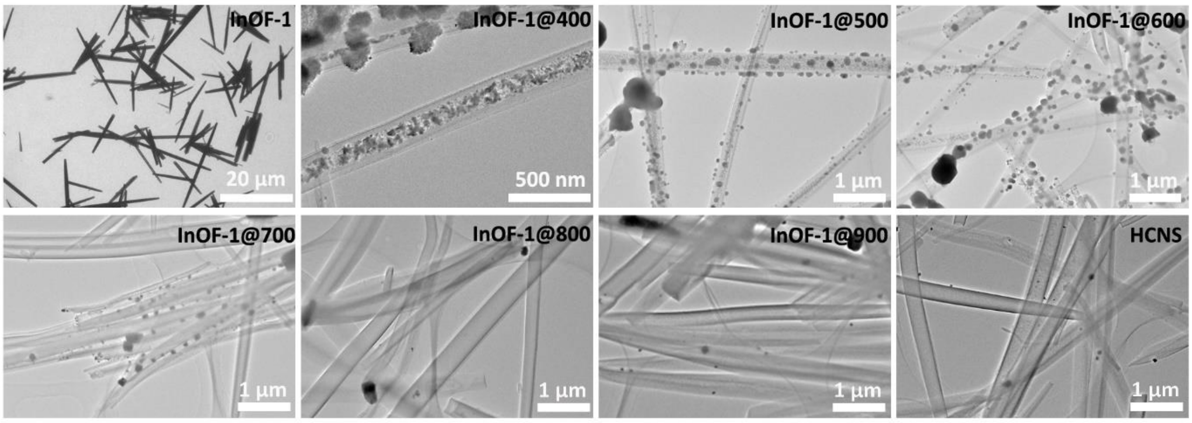

Figure S8. TEM images of initial **InOF-1** nanorods and its derivatives **InOF-1@T** (T=400-1000) annealed at various temperatures.

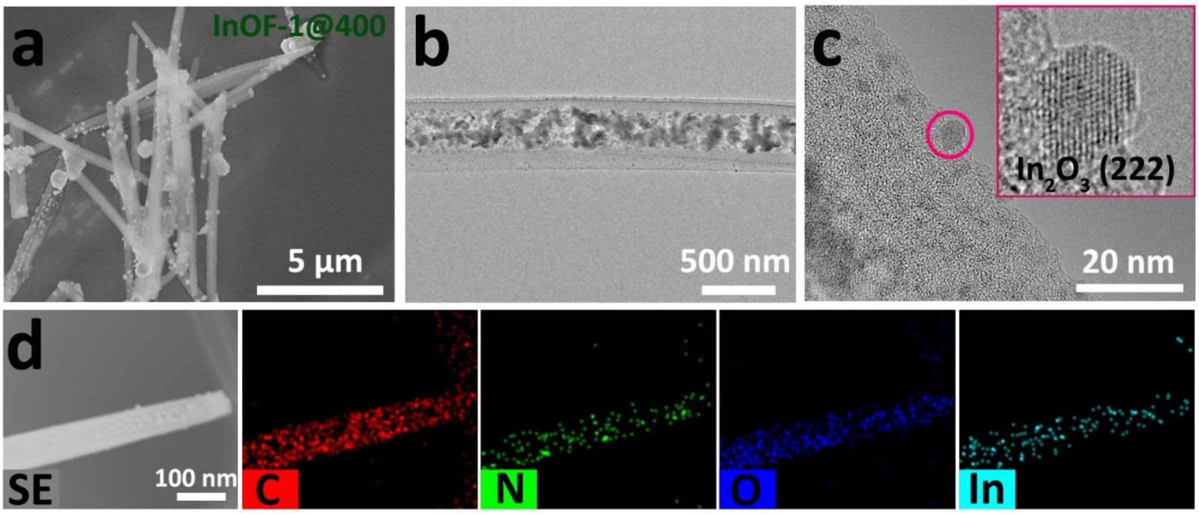

Figure S9. (a) SEM, (b) TEM, (c) HR-TRM images and (d) HAADF-STEM image and EDX element mapping showing the distribution of C, N, O and In of **InOF-1@400**.

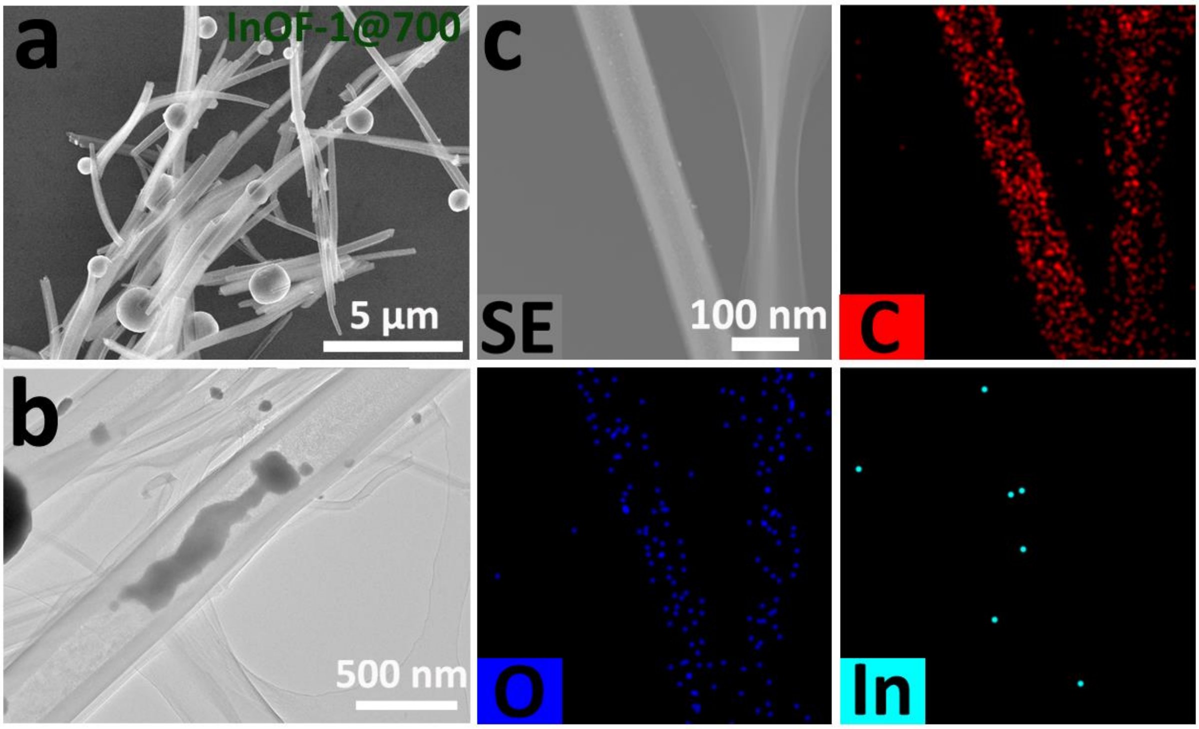

Figure S10. (a) SEM, (b) TEM, (c) HR-TRM images and (d) HAADF-STEM image and the corresponding EDX element mapping of **InOF-1@700**.

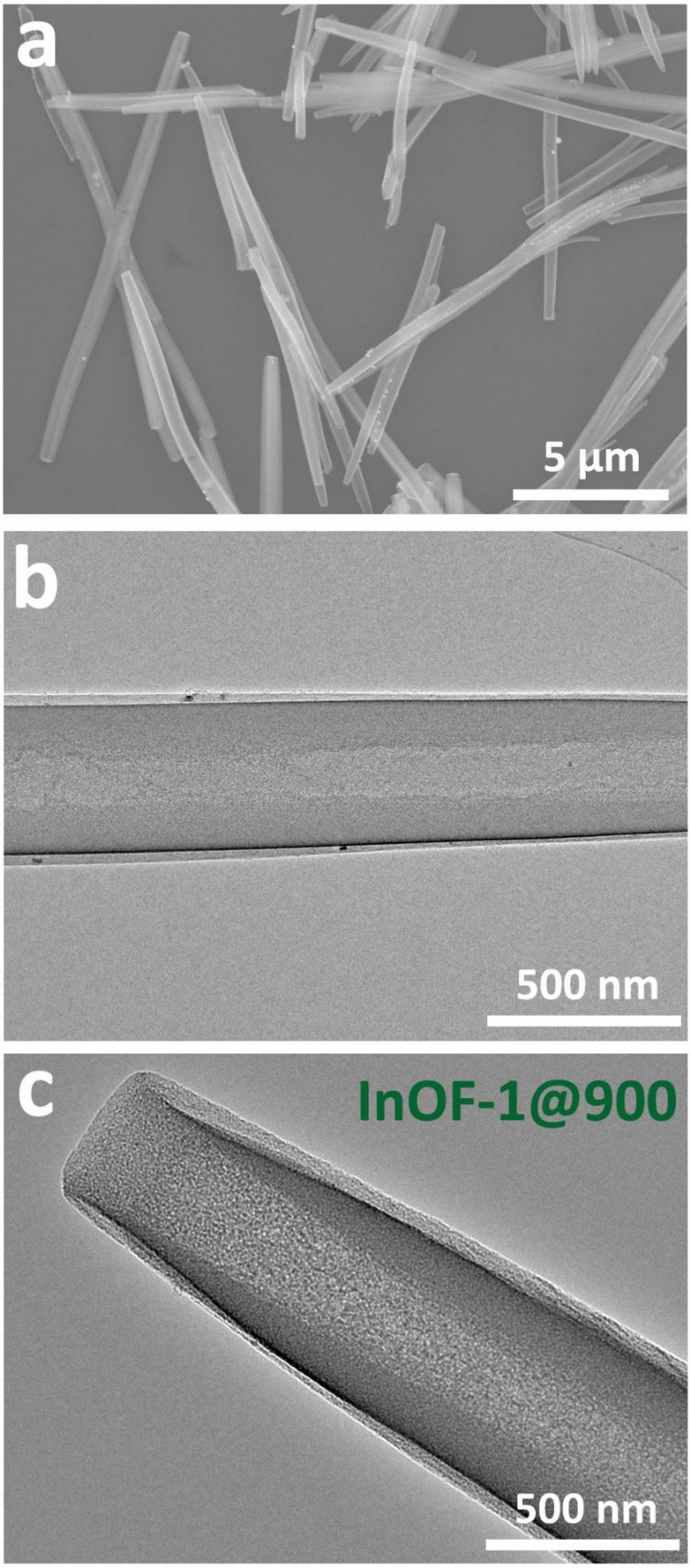

Figure S11. (a) SEM and (b, c) TEM images of **InOF-1@900**.

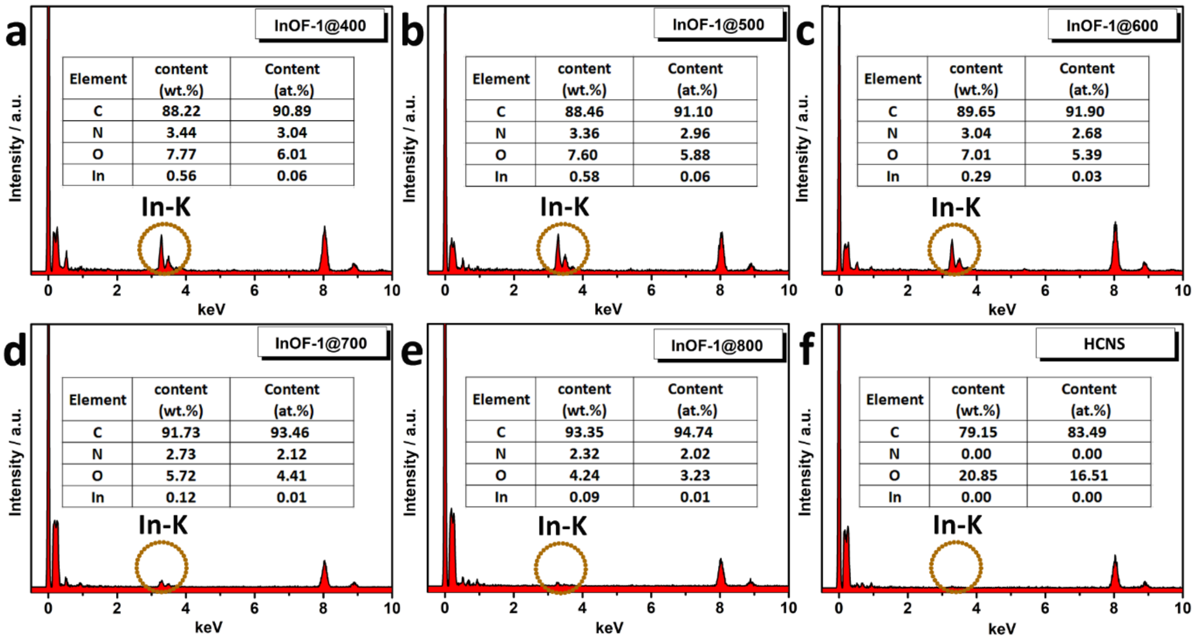

Figure S12. EDX spectra and the corresponding element contents for **InOF-1@400** (a), **InOF-1@500** (b), **InOF-1@600** (c), **InOF-1@700** (d), **InOF-1@800** (e), and **HCNS** (f).

XPS Data

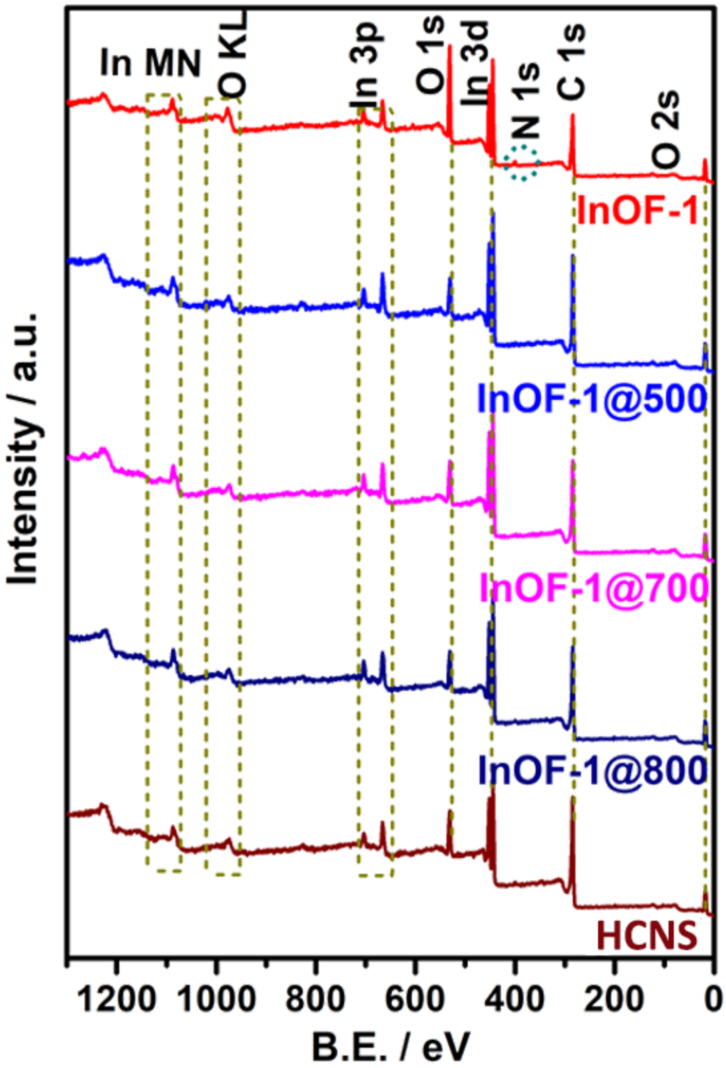

Figure S13. XPS survey spectra of **InOF-1** and its derivatives of **InOF-1@500**, **InOF-1@700**, **InOF-1@800**, **HCNS** annealed at various temperatures.

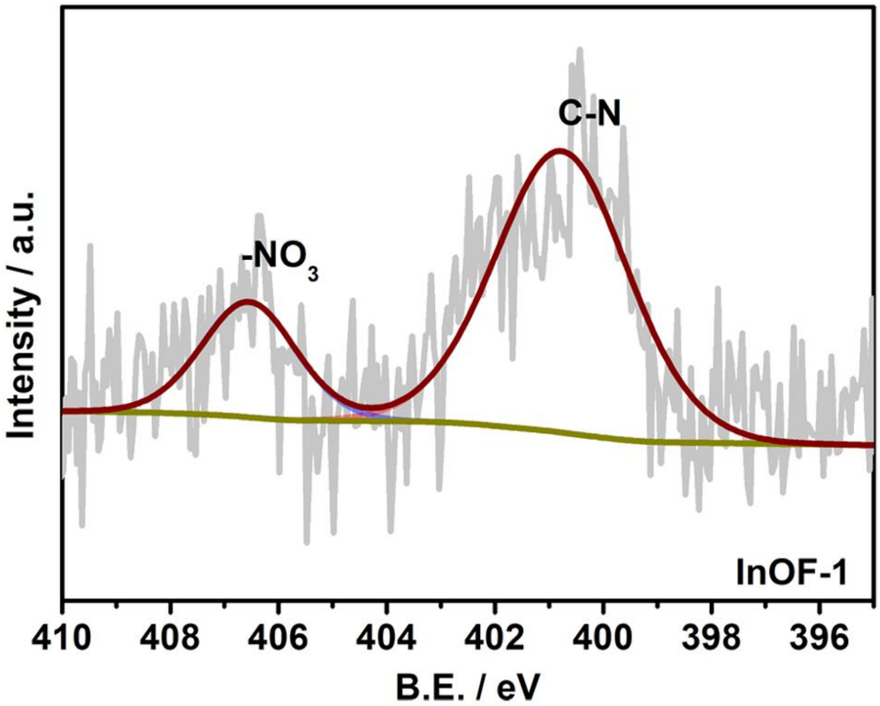

Figure S14. XPS N 1s spectra of **InOF-1**.

Raman Data

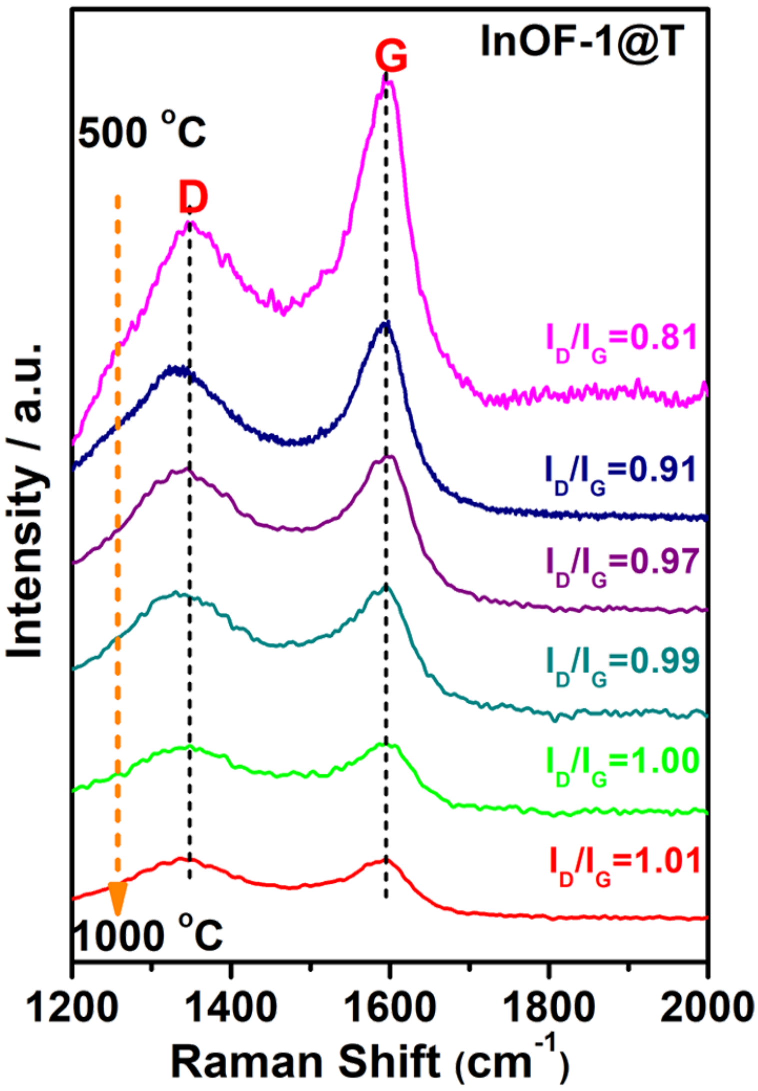

Figure S15. Raman spectra of **InOF-1** and **InOF-1@T** samples.

Sorption Analyses

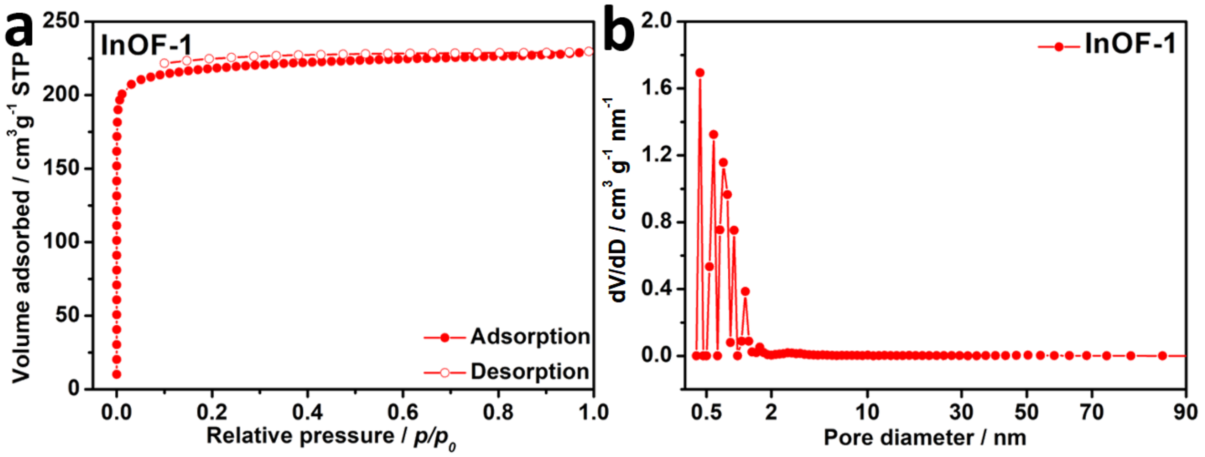

Figure S16. (a) Nitrogen adsorption-desorption isotherms and (b) Pore size distribution calculated using NLDFT method for **InOF-1**.

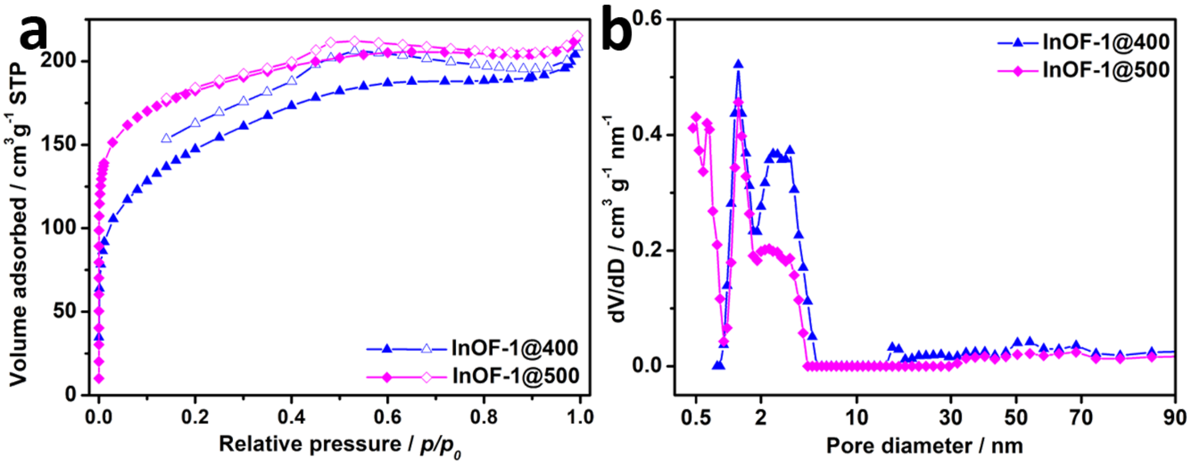

Figure S17. (a) Nitrogen adsorption-desorption isotherms and (b) Pore size distribution calculated using NLDFT method for **InOF-1@400** and **InOF-1@500**.

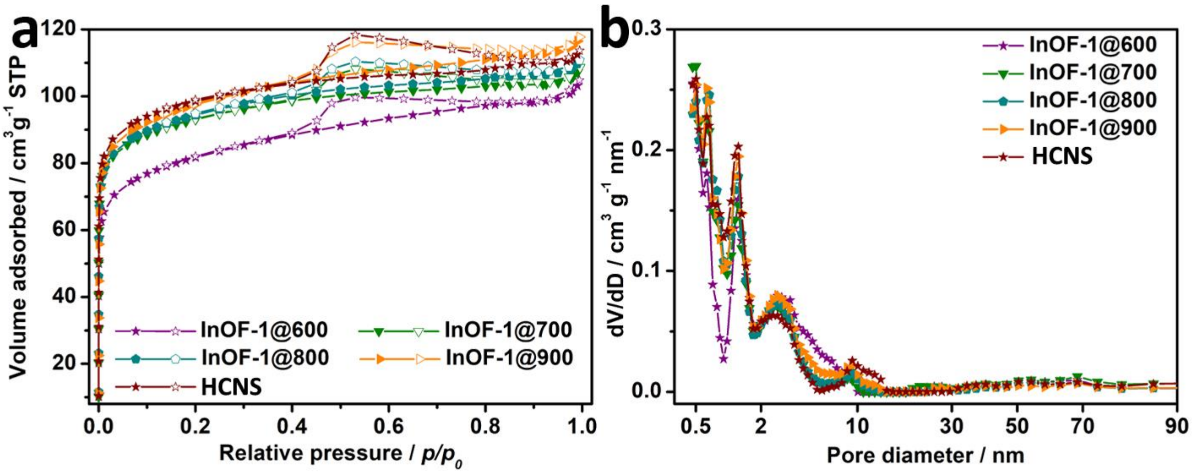

Figure S18. (a) Nitrogen adsorption-desorption isotherms and (b) Pore size distribution calculated using NLDFT method for **InOF-1@600-900, HCNS**.

Table S3. Summary of pore characteristics of InOF-1 and it derivatives of InOF-1@T series annealed at various temperatures.

Sample	Surface area /$m^2\ g^{-1}$		Total pore volume[a] /$cm^3\ g^{-1}$	Micropore volume[b] /$cm^3\ g^{-1}$
	BET	Langmuir		
InOF-1	870.9	937.8	0.36	0.31
InOF-1@400	518.6	538.8	0.31	0.02
InOF-1@500	663.5	762.8	0.33	0.14
InOF-1@600	301.9	326.7	0.16	0.07
InOF-1@700	348.7	382.7	0.17	0.09
InOF-1@800	353.2	388.0	0.17	0.09
InOF-1@900	363.6	399.3	0.18	0.09
HCNS	370.5	406.6	0.17	0.10
CNT	192.9	195.8	0.87	0.01

[a] At P/P_0= 0.99.

[b] Determined by t-plot method.

Table S4. Elemental analyses of HCNS/I_x materials based on EDX results.

Samples	C		O		K		I	
	Weight / %	Atomic / %	Weight / %	Atomic / %	Weight / %	Atomic / %	Weight / %	Atomic / %
HCNS/$I_{0.1}$	48.67	67.98	22.53	23.63	15.44	6.63	13.36	1.77
HCNS/$I_{0.2}$	38.24	59.8	21.97	25.79	25.63	12.31	14.16	2.1
HCNS/$I_{0.5}$	24.39	48.03	19.34	28.59	30.79	18.63	25.48	4.75
HCNS/$I_{0.75}$	19.66	41.8	19.7	31.43	32.25	21.06	28.39	5.71

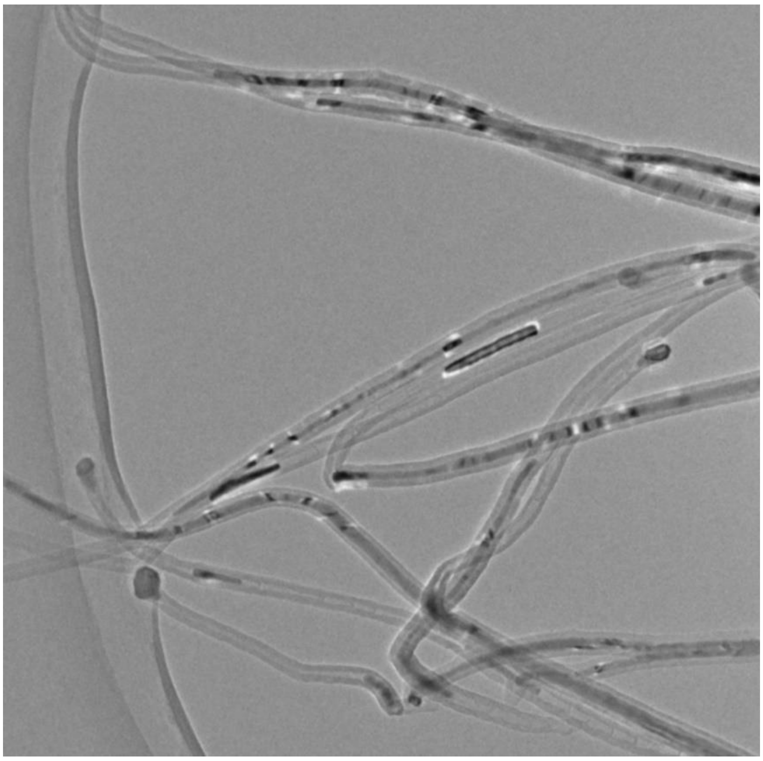

Figure S19. SEM images of CNT/I_x composite.

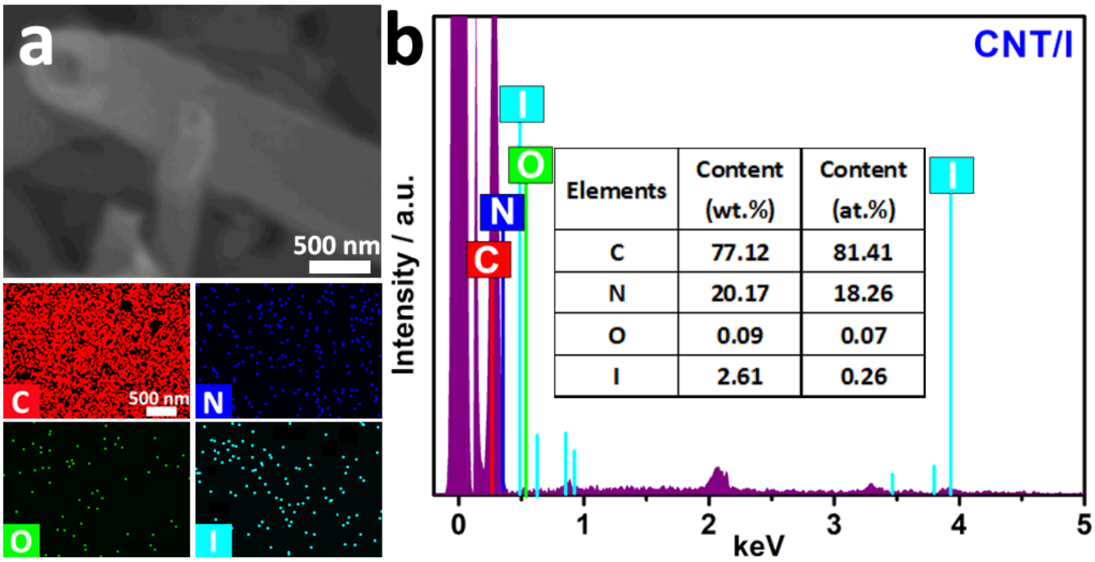

Figure S20. (a) EDX element mapping showing the distribution of C, N, O, and I of CNT/I. (b) EDX spectra and the corresponding element contents of CNT/I.

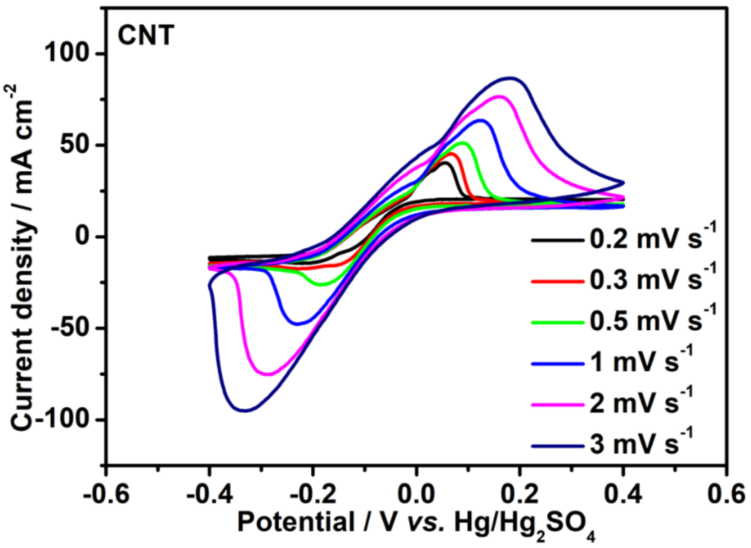

Figure S21. CV curves from 0.2 to 3 mV s^{-1} of **CNT**.

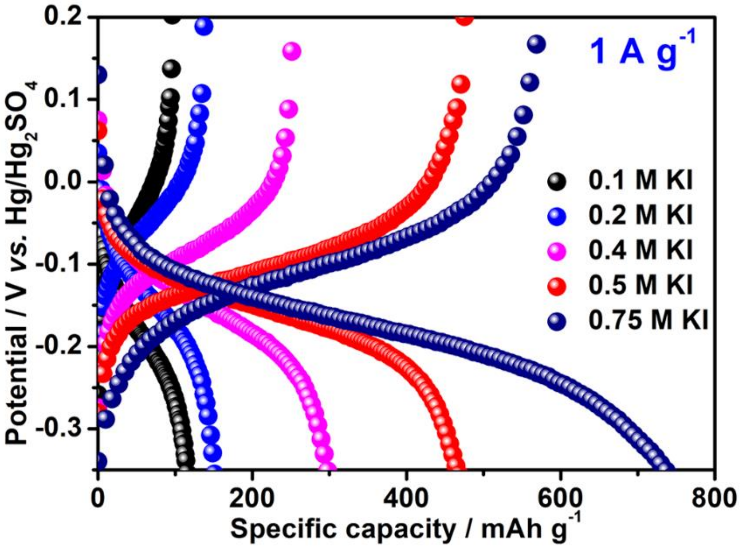

Figure S22. Voltage profiles of **HCNS/I$_x$** at 1 A g^{-1}.

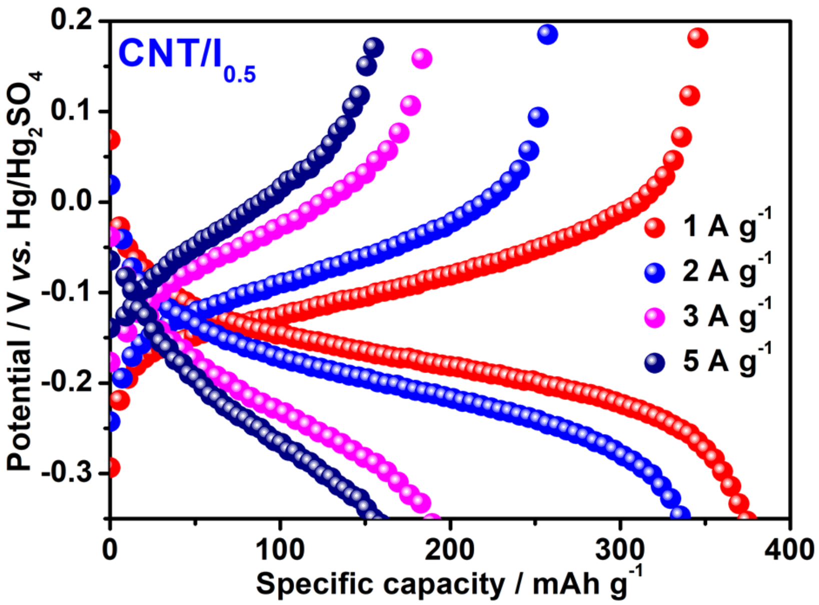

Figure S23. Voltage profiles of CNT/$I_{0.5}$ at various current densities.

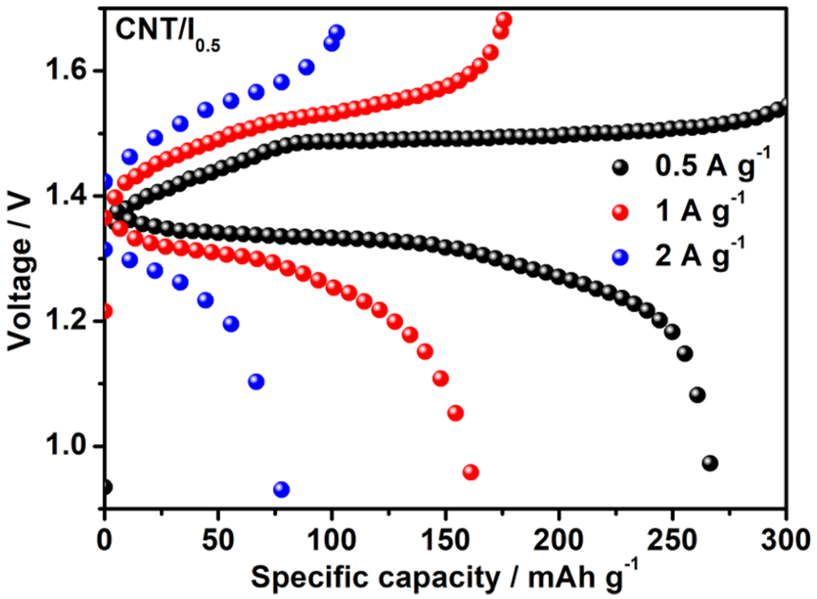

Figure S24. Voltage profiles of a Zn-I_2 battery with a CNT/$I_{0.5}$ as cathode and Zn flake as anode material at different current densities.
